# Montmorillonite-Based Two-Dimensional Nanocomposites: Preparation and Applications

**DOI:** 10.3390/molecules26092521

**Published:** 2021-04-26

**Authors:** Runzhi Wang, Huijie Li, Guangxu Ge, Nan Dai, Jinsong Rao, Haodi Ran, Yuxin Zhang

**Affiliations:** 1 College of Environment and Ecology, Chongqing University, Chongqing 400044, China; runzhiwang2021@163.com; 2 College of Mechanical and Vehicle Engineering, Chongqing University, Chongqing 400044, China; lhjlhj_0804@163.com; 3 College of Materials Science and Engineering, Chongqing University, Chongqing 400044, China; guangxuge@dicp.ac.cn (G.G.); 20200901049@cqu.edu.cn (N.D.); rjs@cqu.edu.cn (J.R.); 4 School of Materials Science and Engineering, Harbin Institute of Technology (Shenzhen), Shenzhen 518055, China; ranhaodi66@163.com

**Keywords:** montmorillonite, two dimensional materials, layered double hydroxide, graphene

## Abstract

Montmorillonite (Mt) is a kind of 2:1 type layered phyllosilicate mineral with nanoscale structure, large surface area, high cation exchange capacity and excellent adsorption capacity. By virtue of such unique properties, many scholars have paid much attention to the further modification of Mt-based two-dimensional (2D) functional composite materials, such as Mt-metal hydroxides and Mt-carbon composites. In this review, we focus on two typical Mt-2D nanocomposite: Mt@layered double hydroxide (Mt@LDH) and Mt@graphene (Mt@GR) and their fabrication strategies, as well as their important applications in pollution adsorption, medical antibacterial, film thermal conduction and flame-retardant. In principle, the prospective trend of the composite preparation of Mt-2D nancomposites and promising fields are well addressed.

## 1. Introduction

Montmorillonite (Mt) is one of the most common 2:1 type clay minerals, which is rich in natural content and non-toxic [1]. Due to its unique structure and surface properties, Mt shows strong water absorption, swelling, dispersibility, good cation exchange performance and adsorption capacity, indicating that it has great potential in removing heavy metal ions and organic cations from sewage [2]. Meanwhile, Mt also presents excellent selectivity and catalytic activity [3], which could contribute to its wide use in organic reactions and controlled release of drugs [4].

Mt also exhibits promising advantages including high thermal stability, high modulus, high strength and low expansion coefficient. However, due to the hydrophilic and oleophobic properities of natural Mt, as well as poor compatibility with polymers, it fails to be widely applied. Therefore, many scholars have modified Mt and enhanced its adsorption and ion exchange properties via preparation with other two-dimensional materials [5], extending the applications in sewage treatment, as a medical antibacterial, in flame retardancy [6] and other fields [7].

Even though some researchers have made progress of the modification of Mt, the preparation of Mt@two-dimensional materials a systematic review on the composite preparation methods and applications of Mt and two-dimensional materials is still lacking [8]. Thus, this review summarizes the preparation methods of two typical composite materials with Mt, including layered double hydroxide (LDH) and graphene (GR) and their practical applications.

## 2. Mt and Its Modification

The layer height of Mt is nearly 1 nm, and the interlayer distance is less than 1 nm, indicating Mt has a nanosized porous structure [9]. Mt is negatively charged [10] with the main components being Si and Al. The well-dispersed particles show a perfect crystal structure [11], which is formed by a two-dimensional layer: an octahedral alumina sheet sandwiched between two tetrahedral silicon wafers [12]. The general formula of Mt can be written as (Al_2-x_Mg_x_)Si_4_O_10_(OH)_2_·(M·nH_2_O) (M: Na^+^, Ca^2+^, and Mg^2+^, etc.). The isomorphic substitution in layers leads to insufficient charge (Fe^2+^ or Mg^2+^ replaces Al^3+^), and the charge is compensated by cations (usually Na^+^ or K^+^) absorbed in an interlayer space of Mt, which leads to its important cation exchange properties [13]. Such a unique structure and surface properties bring Mt great advantages in adsorbing heavy metal cations and cationic dye pollutants.

Highly depending on pH, the polymerization degree of Mt decreases as pH increases before reaching the isoelectric point. Moreover, the charge on edges and surfaces is different [14]. The uniform dispersion of Mt in the polymer matrix serves to enhance its mechanical and physical properties. Organic modification can improve its degree of dispersion and modified layered silicates can be well dispersed in synthetic and biopolymers, showing that the properties can be improved and enhanced to varying degrees. Therefore, Mt composites have broad application prospects in many fields [15].

Currently, the modifications of Mt are mainly divided into three categories, including organic modification, inorganic modification, and organic-inorganic composite modification. For organic modification, cations in the interlayer space are exchanged by the ions with a functional group of surfactant molecules, which act as an interlayer opener [16]. Organic modification is beneficial to increase the compatibility between polymers and Mt, to obtain composites with enhanced properties [17]. Some researchers have used intercalation methods [18] to prepare organically modified Mt which showed a good dispersion level and improved thermo-mechanical properties [19]. However, the interaction between modifiers and Mt surface has not been thoroughly studied and systematically clarified. Greesh et al. [20] modified sodium-based Mt with 2-acrylamide-2-methylpropanesulfonic acid (AMPS) and *n*-isopropylacrylamide (NIPAm) to explore which chemical group was the driving force for AMPS adsorption in the surface of Mt, and finally revealed that it was the main interaction between AMPS and the clay [21]. In 2015, Kumar and Kannan [22] found that by controlling the free radical polymerization time, the amount and molecular weight of the polymer in Mt interlayer space can be easily modulated, realizing the controllable operation of intercalation and exfoliation of Mt. The abovementioned studies show that it is both feasible and beneficial to improve the dispersibility of Mt by organic modification.

Inorganic modification is widely used for preparing Mt-based 2D composites. To enhance the adsorption capability of natural Mt for heavy metal ions, as early as the 1870s, inorganic methods were proposed to modify Mt, such as acid activation modification, inorganic salt modification and pillared modification. These methods can increase the interlayer distance and strengthen the thermal stability of Mt for using it as the adsorption material to remove heavy metal ions [23]. Cheng et al. [24] prepared Mt nanoclay/maleic anhydride modified polypropylene composites (PPCNs) by acid modification, in which the existence of nanoclay effectively changed the thermodynamic properties of polypropylene. Besides, Chen et al. [25] found that Mt modified by sodium group can adsorb heavy metal ions such as Pb^2+^, Cd^2+^, Cu^2+^, Co^2+^, etc., it can quickly reach equilibrium within 10 min. Indeed, the performance of Mt, especially in terms of thermal stability and adsorption capacity of heavy metal ions, has been improved through inorganic modification.

Besides, the organic-inorganic modification has also been used. Surfactant modification can greatly improve the adsorption selectivity of Mt for heavy metal ions, while it blocks the interlayer space of Mt, reducing the porosity and the adsorption capability of pollutants. However, pillared Mt can significantly increase the pore volume and surface area and has stronger tolerance to pH and coexisting inorganic ions. Therefore, preparing inorganic-organic composite modified Mt contributes to improving its adsorption capability for pollutants [26].

## 3. Two-Dimensional Materials

The concept of two-dimensional materials was proposed in 2004, when scholars at the University of Manchester successfully prepared single-layer graphene by mechanical stripping method [27]. Two-dimensional materials refer to materials in which electrons can only move freely on the nanoscale of two dimensions (1–100 nm).

According to the composition and structure of materials, the existing two-dimensional nanomaterials can be divided into five categories:

(1) Simple substances: graphene, graphdiyne, black phosphorus (BP), metals (Au, Ag, Pt, Pd, Ir, and Ru) and new boron, arsenic, germanium, silicon, bismuth, and so on.
(2) Inorganic compounds: hexagonal boron nitride (h-BN), graphite phase nitrogen carbide (g-C_3_N_4_), boron carbon nitrogen, and various graphene derivatives.
(3) Metal compounds: transition metal disulfide (TMDs), Layered double hydroxide (LDH), transition metal oxide (TMOs), transition metal carbon/nitrogen/carbonitride (MXenes), metal phosphorus trisulfide APX_3_, metal halide, transition metal oxyhalide (MOX), III-VI layered semiconductor (MX).
(4) Salts: inorganic perovskite compound (AMX_3_) clay mineral (layered aluminosilicate containing water).
(5) Organic frameworks: layered metal-organic framework compounds (MOFs), layered covalent organic framework compounds (COFs) and polymers [28].

Due to the limitation of electrons in two dimensions, two-dimensional features are unique and necessary for obtaining unprecedented physical, electronic and chemical properties [29]. Compared with traditional materials, the unique properties of two-dimensional materials include:

(1) Ultra-high mechanical strength. Two-dimensional materials have strong fracture resistance, good toughness, and are ductile but not easy to break [30].
(2) Good electrical properties. On the one hand, electrons are restricted to the limited domain of two-dimensional materials with no interlayer interaction, which can greatly stimulate the electronic properties; on the other hand, the large transverse dimension and ultra-thin thickness give them extremely high specific surface area, exposing more active sites on the surface to the greatest extent, thus they are widely used in catalysis or energy storage fields [31].

At present, many researchers have explored efficient preparation methods for two-dimensional nanomaterials [32]. The recognized methods include: micromechanical dissociation [33], mechanical force-assisted liquid peeling, ion insertion-assisted liquid exfoliation [34], ion exchange-assisted liquid exfoliation and so on [35]. All methods can be divided into two categories: top-down methods and bottom-up methods. Ultra-thin two-dimensional nanomaterials prepared by different synthesis methods may exhibit different physical, electronic, chemical, and surface properties. It is important to measure the exact parameters and to understand the correlation between structural characteristics and performance (such as size, composition, thickness, crystal phase, doping, defect, vacancy, strain, electronic state and surface properties) [36].

Among two-dimensional nanomaterials, GR is the most powerful nanomaterial and has been widely used in many fields [37]. Meanwhile, as a new anionic clay material, LDH has broad application prospects in flame retardancy [38], pollutant adsorption [39], catalytic synthesis [40], medical treatment and other fields, raising the demand for it in recent years [41]. Based on the excellent properties and good application prospects of GR and LDH, this review introduces the preparation and applications of these two typical two-dimensional materials-Mt composites respectively, which could be extended to other composites with two-dimensional materials.

## 4. Preparation and Application of Mt@LDH

Layered double hydroxide (LDH), also known as anionic clay or hydrotalcite-like, is a kind of mineral material composed of two or more metal elements, which is similar to the octahedral brucite layered structure. It has the characteristics of adjustable metal composition, interlayer anion, size and thickness of the laminate, etc. [42]. The unique structure of LDH makes it useful in catalysis [43], as a catalyst carrier [44], in ion conversion [45], flame retardant materials [46] and other fields. However, the thermal stability of LDH is poor, and its applications are limited due to its multi-layered stacked structure, small specific surface area, insufficient exposure of catalytic active sites existing between its layers and so on. However, Mt has larger specific surface area, higher hydroxyl removal temperature and better thermal stability, therefore, combining their respective characteristics to complement each other has become an important research direction [47]. In terms of adsorption, Zhang et al. [48] have tried LDH modification to improve the adsorption rate by 18.3% [48], which was an improvement but was still not ideal. Therefore, many scholars have combined anionic clay mineral LDH with cationic clay mineral Mt, using Mt’s higher specific surface area, good thermal stability, and lamellar structure to improve the structural characteristics of LDH. The results showed that the combination of LDH and Mt can indeed improve the structural characteristics of LDH, and the application areas of LDH were expanded significantly. In the past studies, the general methods to realize the recombination of Mt and LDH included: the intercalation method, in-situ growth method, tape casting method and other methods.

### 4.1. Preparation of Mt@LDH

#### 4.1.1. The Intercalation Method

**Figure 1:**
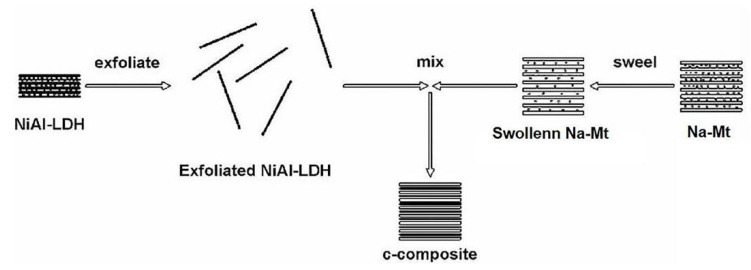
Composite materials synthesized by an intercalation method [49].

The main content of intercalation assembly method is that LDH and Mt are exfoliated and intercalated to obtain composite materials. When the exfoliated LDH nanosheets are intercalated with Mt, the swelling property and high specific surface area of Mt in water can be used to increase the exposure of the inner space of LDH layer. In this way, the layered stacking structure of LDH can be changed to increase the specific surface area, and finally the composite material with chip assembly structure can be obtained. The process of synthesis of NiAl-LDH/Mt composites by Zhou et al. [49] is illustrated in Figure 1.

There are two key challenges in the preparation of composite materials by intercalation methods. One is how to develop an effective method to peel off the laminated composite completely, and the other is how to adjust and control the assembly of nanosheets to obtain the nanocomposite with specific structure [50]. Before stripping, some methods require the materials to be modified in advance, so as to facilitate better stripping of the sheets in the stripping agent [51].

Mt and LDH are closely aligned due to the interaction between interlayer ions and laminates, so stripping Mt and LDH to obtain stable sol is a prerequisite for preparing thin film materials [52]. Most scholars chose to modify Mt and LDH, and then peel them off by an ultrasonic method (Table 1).

According to the different stripping agents used, the LDH stripping methods can be divided into three types: stripping in short chain alcohols, stripping in formamide and stripping in water. Stripping in polar solvents such as formamide is considered to be one of the simplest and most efficient methods, which makes formamide one of the most common stripping agents [49].

**Figure 2:**
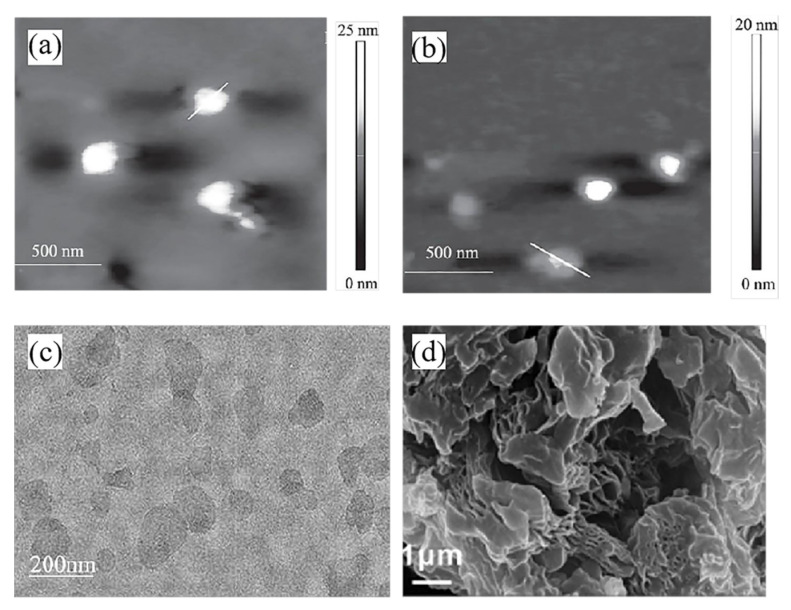
Images of lamellar stripping products of two-dimensional materials. (**a**) AFM image of exfoliated CTA-Mt [55], (**b**) AFM image of exfoliated MgAl-SDS-LDH [55], (**c**) TEM image of stripped Aco-LDH nanosheets [56], (**d**) SEM image of exfoliated LDH [50].

In addition, using chloroform as stripping agent is also common. Gong [54] and Dai et al. [55] both used chloroform as stripping agent. In the latter study, CTA-Mt (organic montmorillonite) and MgAl-SDS(sodium dodecyl sulfonate)-LDH can be stripped in chloroform medium in only 20 min, which greatly reduces the time required for lamellar stripping. Electron micrographs of the stripped product are illustrated in Figure 2a,b.

However, the use of the above two stripping agents has certain limitations or potential hazards. The strong corrosive environment of the formamide stripping system limits the application of the stripped nanosheets, and the formamide solution is not volatile, which is not conducive to the synthesis of composite materials. Meanwhile, chloroform is sensitive to light. When exposed to light, it can react with oxygen in the air and gradually decompose into highly toxic phosgene, which is not only harmful to the environment but also dangerous in the preparation process. Therefore, some scholars put forward the stripping of LDH and Mt in water or other cheap degradable agents, to realize the economical and environment-friendly stripping.

Due to the strong polarity of water molecules between LDH layers, interlayer anions with hydrophilic groups can improve the swelling performance of LDH in water. To achieve a more thorough stripping, it is necessary to balance the size of intercalated anions, as well as their relationship with hydrophilicity and hydrophobicity between laminates. Acetate, as a common short-chain carboxylate, can better meet the requirements for swelling and stripping of intercalated LDH in water [59]. To synthesize NO_3_-LDH by a hydrothermal method, Liang et al. [56] obtained LDH intercalated with acetate ions by an ion exchange method, and then studied its stripping behavior in water by ultrasonic dispersion and other technical methods (a TEM image of exfoliated Aco-LDH nanosheets is shown in Figure 2c). Then, they intercalated the exfoliated LDH nanosheets with Na-Mt, and made use of the swelling property and high specific surface area of Mt in water to increase the exposure of the inner space of LDH layer, and changed its layered stacking structure to increase the specific surface area, finally obtaining a composite material with a sheet-to-sheet assembly structure.

Ma et al. [57] prepared hydrotalcite-like compound/Mt inorganic layered composites in water systems. They used the properties of the amino acid isoelectric point to synthesize amino acid-intercalated Al-Mg hydrotalcite in a system with pH greater than amino acid isoelectric point.

Kojima et al. [60] found that when Mt is exfoliated in a polymer, a real nanocomposite can be formed, which has excellent physical and mechanical properties. Ginzburg and Balazs [61] found that polymers containing terminal functional groups are beneficial to exfoliate Mt. Zhu et al. [58] carried out the exfoliation of Mt in HTPB (hydroxyl terminated polybutadiene), which basically realized the complete exfoliation of Mt.

**Figure 3:**
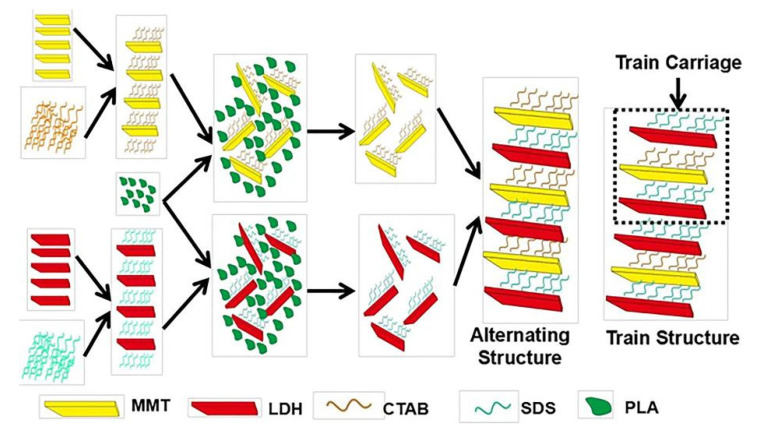
Schematic illustration of the preparation process of Mt@LDH nanocomposite [50].

In addition, some researchers used biodegradable materials such as polylactic acid (PLA) for lamellar peeling. Hu et al. [50] completely and effectively exfoliated LDH and Mt in PLA by melt blending for 10 min, and the resulting exfoliated monolayer molecules of Mt or LDH could be stably stored in PLA for a long time (SEM image of exfoliated LDH is illustrated in Figure 2d). Table 1 summarizes common delamination methods. Besides, melt peeling can also be performed by Mt lamellar peeling [9]. In this process, Mt is first mixed with thermoplastic polymer, and then the mixture is heated under the action of shear force. If there is enough polymer compatible with the surface of the layer, the polymer molecular chain can also enter the interlayer space to form polymer intercalated-Mt or exfoliated Mt/polymer nanocomposite. In the melting process, the enhancement of mechanical force will increase the mobility and diffusion of polymer, improving the dispersion and peeling of Mt in polymer matrix. The preparation process of Mt@LDH nanocomposites is illustrated in Figure 3.

Because Mt and hydrotalcite are similar in structure, the charges between layers are completely opposite. These characteristics provide natural conditions for the combination of Mt and hydrotalcite through electrostatic interaction, which have been used to combine Mt lamellae with LDH lamellae.

Liang et al. [56] first dispersed acetate layered double hydroxide (Aco-LDH) in deionized water, magnetically stirred the suspension for a certain time, then ultrasonicated it in a room temperature water bath for 1 h and centrifuged it at high speed to isolate the upper translucent colloid solution. After dispersing and pretreating Mt in deionized water, the colloidal solution is added, and magnetic stirring is carried out at room temperature, so that Mt is fully dispersed and swelled. Aco-LDH nanosheets intercalated into the interlayer, thus obtaining the composite material.

Chen et al. [62] added a third substance to prepare the laminated composite structure in order to further improve the composite effect of peel-off laminates. They prepared CTA-Mt by an ion exchange method and MgAl-SDS-LDH by a hydrothermal method. Using ethyl acetate as stripping medium, a stable sol was obtained by ultrasonic stripping, and then PVA/Mt/PVA/LDH thin films were prepared by assembling polyvinyl alcohol (PVA) and inorganic nanosheets layer by layer.

#### 4.1.2. In-Situ Synthesis Method

In-situ synthesis is a novel method to prepare composite materials. The basic principle is that different elements or compounds react chemically under certain conditions, and one or several ceramic phase particles are formed in the metal matrix, to improve the performance of single metal alloy. The reinforcement nucleates and grows spontaneously in the metal matrix, so there is no pollution on the surface of the reinforcement. The compatibility between the matrix and the reinforcement is good, and the interfacial bonding strength is high. Compared with other composite materials, the complicated pretreatment process of reinforcement is omitted, and the preparation process is simplified.

**Figure 4:**
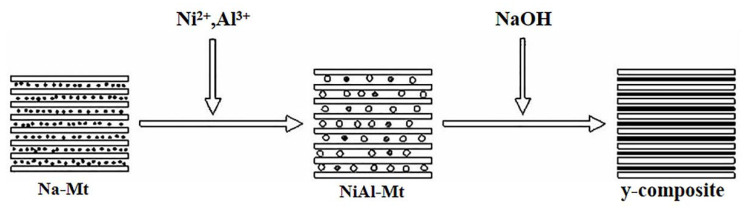
Preparation of composites by in-situ composite method [49].

Zhou et al. [49] pointed out that NiAl-Mt was first prepared through Na-Mt ion exchange, and then the NiAl-Mt obtained above was put into deionized water and stirred evenly to obtain NiAl-Mt slurry. Finally, the NiAl-LDH/Mt composite material was prepared in situ. The process of obtaining NiAl-LDH/Mt composites by in-situ synthesis is illustrated in Figure 4.

They also discovered that the specific surface area (10 m^2^/g) of the composite obtained by intercalation method was quite different from that obtained by in-situ method (168 m^2^/g), which indicated that the internal structure of the composite obtained by the two methods was different.

#### 4.1.3. Tape-Casting Method

Casting is a production technology for plastic thermoformed sheets, which can be used to process single-layer or multi-layer plastic thermoformed sheets of polypropylene (PP), polystyrene (PS) and high impact polystyrene (HIPS). The tape casting process has the advantages of a convenient process, adjustable size and good film forming quality [63]. It is not common in the combination of Mt and LDH, and is usually used in the preparation of composite membranes. Xu et al. [64] first prepared a PLA/Na-Mt/MgAlCu-LDH ternary composite coating by a solution blending method. They coated the prepared series of film coating solutions on the polytetrafluoroethylene plate using a tape casting method through an automatic film coater. This whole body was naturally dried for 24 h, then put into a vacuum drying box for drying, and was then removed after film formation. Finally, PLA/Na-Mt/MgAlCu-LDH was prepared. The ternary composite membrane achieved better thermal stability, tensile strength, and elongation at break using the proper proportion of ingredients.

In the process of compounding Mt with LDH, there are three existing routes: in-situ synthesis methods, intercalation methods and melting methods. Most scholars use intercalation methods to combine the two materials. These can be divided into two steps: delamination and composition. Formamide and chloroform are the most common stripping agents used in lamellar stripping, and their stripping steps are quite simple and efficient. However, in some cases, they have certain use restrictions or represent potential hazards. Therefore, some scholars have been working on the use of water as stripping agent, as well as other environmentally friendly degradable materials.

### 4.2. Applications of Mt@LDH

#### 4.2.1. Pollutant Adsorption

Heavy metal pollution can affect human health through the food chain [65], so how to control heavy metal pollution has become a research hot spot. Although the adsorption method is simple in operation and low in cost [66], it has some disadvantages, such as small adsorption capacity, poor adsorption stability and difficult separation after use. Therefore, the development of adsorption materials with large adsorption capacity, good stability, and easy separation is needed.

LDH, with anion exchangeability, has attracted much attention due to its unique properties in the removal of pollutants from water [67]. On the other hand, Mt has a large surface area. Hence, some scholars combined them to combine their respective advantages and enhance the adsorption effect [68].

Bakr et al. [69] prepared a LDH adsorbent through coprecipitation method, and prepared composites by physical mixing of LDH and Mt by high shear. Mn^2+^ and other heavy metal ions in water were adsorbed and thus removed. The experimental results proved that the adsorption effect was improved with the increase of temperature and the prepared Mg-Zn-Al (LDH)@Mt had better adsorption effects than LDH and Mt alone. Shehap et al. [70] reached the same conclusion and Bakr et al. [71] also found that the optimum adsorption capacity was obtained at pH 6 and T = 318 K after 105 min in the presence of 0.25 g of Mg-Zn-Al (LDH)@Mt.

Wang and Li [72] found that the maximum adsorption capacity of phosphate on Mt@LDH was 127.8 mg/g, about 21% higher than that of pure LDH. They also suggested that the enhanced adsorption of phosphate by Mt@LDH might be due to its structural change compared with the precursor. The results showed that there was a synergistic effect between LDH and Mt during phosphorus removal.

Seddighi et al. [73] synthesized Mt@LDH by an in-situ growth method. Characterization and analysis showed that clay-based nanocomposites with double adsorption properties had been successfully prepared. LDH grew on the surface of Mt. In the presence of carbohydrate complex ligands, U(VI) adsorption capacity was significantly improved. The kinetics showed that more than 95% of uranyl ions were adsorbed within 30 min. A cost-effective and environment-friendly adsorbent is proposed, which can be used to remove uranyl carbonate species from natural and sewage. Jiang et al. [74] also performed relevant studies on the adsorption performance of Mt@LDH. They successfully prepared Mt layered dihydroxy nanosheets by a one-pot hydrothermal method. The anionic dye methyl orange (MO) and cationic dye methylene blue (MB) were removed by using 2D-2D growth Mt@NiFeLDH as adsorbent. Under the optimum adsorption conditions, the maximum adsorption capacity of MO and MB by the composite was 108.80 mg/g and 99.18 mg/g, respectively. The adsorption kinetics of MO and MB were in accordance with the pseudo-second-order model. This study provided a simple way to synthesize ideal cationic and anionic dye bifunctional adsorbents, broadening the potential applications of practical wastewater purification.

Due to their remarkable chelate effect on heavy metal ions, hydrotalcite-like materials have potential applications and economic advantages as water purification agents, which is a new research direction in recent years. Therefore, it is meaningful to further optimize the properties of the hydrotalcite and expand its application fields.

#### 4.2.2. Acid-Base Bifunctional Catalysis

Most industrial chemical reactions are carried out under the action of catalysts. Because catalysts have different degrees of activity under different conditions, it is very important to choose appropriate and efficient catalysts in industrial production. Although some reactions are catalyzed by acid sites on the catalyst surface, the base sites play a synergistic role to some extent in reactions such as alkylamine decomposition, aldol condensation and the Knoevenagel reaction, etc. [75]. This discovery aroused the enthusiasm of scholars to study acid-base bifunctional catalysts. The so-called acid-base dual function means that it not only plays the role of an acid site, but the synergy between them is much better. As a catalyst, Mt is mainly used for acid-catalyzed reactions, and LDH laminates have basic sites, which means Mt@LDH is very likely to have base catalytic ability [76]. Additionally, the thermal stability of LDH catalyst is one of the cores to ensure its catalytic activity and life [77]. Therefore, some scholars have prepared Mt@LDH as new acid-base catalysts by using their catalytic properties for acid-base reaction.

Pu et al. [78] and Li et al. [79] realized the technological innovation in the catalytic field by assembling a Mt@LDH with amino acid composite, which has acid-base bifunctional catalysis. An acid-base bifunctional catalyst is a kind of catalyst which can be used for both acid catalysis and alkali catalysis. For complex chemical reactions involving acid-base amphoteric catalysis, if traditional acid-base catalysts are used, neutralization reaction will occur due to the acid and base meeting. When an acid-base bifunctional catalyst is used, due to the active sites which can catalyze acid-base, the active sites do not interfere with each other. That means acid-base neutralization will not occur. Therefore, the complex chemical reactions involving only acid-base catalysis are expected to be completed in one pot under the action of acid-base bifunctional catalysts. This can greatly improve the conversion rate and yield of the reaction and greatly reduce the discharge of waste from the reaction [80,81].

#### 4.2.3. Corrosion Resistance

Due to metal corrosion, discarded and unusable metals account for 15% of the output in the world, while the steel equipment scrapped due to corrosion is equivalent to about 30% of the annual output [82]. To improve this situation, scholars in the field of anticorrosion have studied coatings with superior anticorrosion performance for a long time. Norio [83] found that the corrosion deposited film of a corrosion resistant alloy had ion selectivity. The outer layer facing the environment and the inner layer facing the metal had cation selectivity and anion selectivity, respectively. The structure prevented the invasion of external ions such as chloride ions and the outward migration of metal cations, and promoted the formation of a dense anhydrous oxide inner layer, which promoted the passivation of metal and protects metal. The characteristics of bipolar coatings are that the primer has anion exchange membrane properties, while the topcoat has cation exchange membrane properties [84]. Mt has cation exchange ability [85], and hydrotalcite has interlayer anion exchange property, which lays a foundation for the formation of bipolar coatings.

Some researchers found that ion selective coatings had high protective performance [86]. Dong et al. [87] pointed out that the impedance modulus of a bipolar coating was about two orders of magnitude higher than that of a cationic coating and varnish. In addition, the hardness and adhesion of bipolar coating were higher than those of other coatings. The composite material of LDH and Mt also enriched the studies of bifunctional catalysis.

Dong et al. [88] added LDH and Mt nanoparticles to an epoxy resin coating to study their effects on coating properties. Six kinds of composite coatings were prepared and soaked in NaCl solutions with a mass fraction of 3.5%. The results showed that Mt@LDH coating had the best protective performance among all coatings. The mechanism of improved corrosion resistance of Mt@LDH may be due to cation selectivity of surface layer and anion selectivity of primer, and the existence of nanoclay prolonged the migration path of water, oxygen, and ions.

Interestingly, bipolar anti-corrosion materials have been gradually developed, which also provides new ideas for the further development of anti-corrosion materials. Besides, we can find that Mt@LDH may have the following promising applications:

(i) Sustained drug release. Mt can prevent gastrointestinal discomfort caused by other drugs and has a very high positioning ability. After oral administration, Mt can evenly cover the whole surface of the intestinal lumen and last for 6 h. As Mt is not easily absorbed after oral administration, it does not enter the blood circulation, so it is very safe during the application period and has no toxic reactions. Magnesium aluminum hydrotalcite (MgAl-LDHs) has good biocompatibility. In summary, Mt and LDH with layered structure are two clay minerals that can accommodate a large amount of drugs between the layers, making them the perfect choice for preparing drug sustained-release carriers [89]. Chen [90] has studied the application of Mt and hydrotalcite in new drug delivery systems, and clarified the interaction mechanism between a series of Western and traditional Chinese medicine active ingredients and mineral drugs (Mt, hydrotalcite, etc.). The drug adsorption characteristics and sustained-release theory of new inorganic layered minerals included: (1) the mode of existence of drugs in layered inorganic minerals was related to the size of drug molecules and the type of functional group. (2) The complexes all had characteristic infrared absorption peaks corresponding to various drugs. (3) The complexes contained the same organic functional groups as the drugs. Hydrogen bond existed between that drug and the inorganic layered mineral.

Kevadiya and Bajaj [91] have also developed the use of Mt as a drug delivery carrier to achieve fixed-point release of drugs in the body. However, there was still the problem of low intercalation rate. This was due to the competition between the cationic drug in Mt and the exchangeable H^+^, which reduced the uncharged PA (procainamide hydrochloride) species to below pH 4, destroying the environment. Hydrotalcite has a certain buffering performance.

(ii) Flame retardant effect. Mt and LDH combined with other materials respectively have the effect of improving thermal stability [92]. When hydrotalcite itself is used as a flame-retardant material, it has outstanding advantages such as being halogen-free, non-toxic, no toxicity and corrosive gas formation, with excellent flame retardant and smoke suppression properties, and has become a hotspot in the field of flame retardant research [93]. Mt, which can maintain or even significantly improve mechanical properties while improving flame retardancy, has attracted increasing attention [94].

The advantages and disadvantages of the two materials complement each other, and the composite material should show a greater improvement in flame retardancy. However, this field is currently less studied in academia, and it is worthy of further researches in the future.

## 5. Preparation and Application of Mt@GR

**Figure 5:**
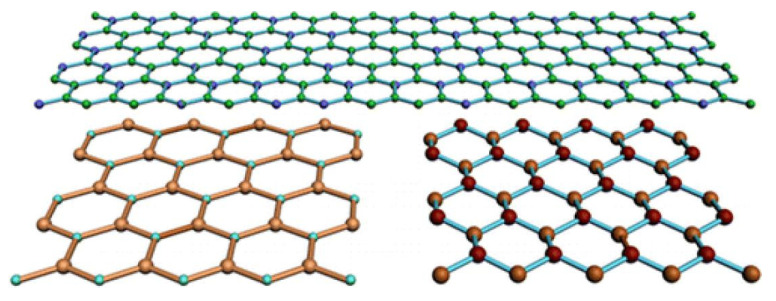
Schematic diagram of the graphene structure [96].

Graphene, also known as “single-layer graphite sheet”, refers to a dense layer of carbon atoms wrapped in a honeycomb crystal lattice (Figure 5). The carbon atoms are arranged in a two-dimensional structure, similar to the monoatomic layer of graphite. It is the only two-dimensional free state atomic crystal discovered so far [95], and it is also the basic building block of a series of famous carbon materials such as three-dimensional graphite, one-dimensional carbon nanotubes, and zero-dimensional fullerenes [96].

As the thinnest substance known in the universe [97], graphene has an extraordinary specific surface area (2630 m^2^/g) [98], ultra-high strength (130 GPa) [99], outstanding thermal conductivity (5000 W/(m·K)) [100], electrical conductivity [101,102] and other excellent properties [103]. In addition, graphene can be easily obtained from the abundant graphite on the Earth’s surface, making it one of the most accessible materials in basic research [104]. Based on the above advantages, graphene materials have been favored by many researchers and become a research hotspot [105].

**Figure 6:**
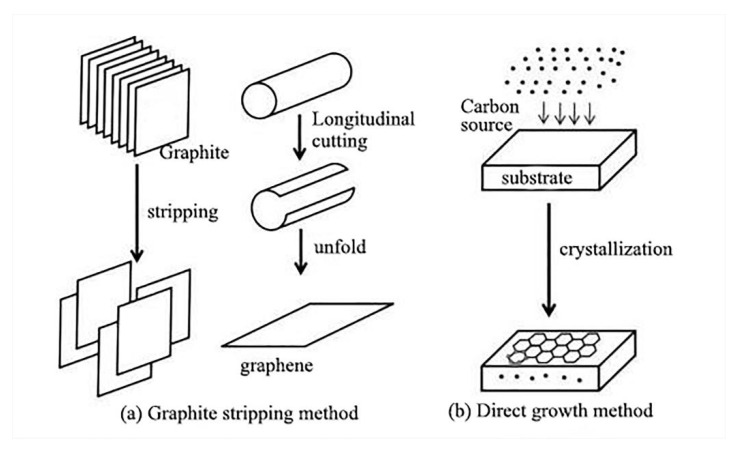
Preparation of graphene [117]. (**a**) The graphite stripping method, in which graphene is obtained by lamellar stripping using graphite or carbon nanotubes as raw materials, and (**b**) the direct growth method, in which carbon sources are introduced under certain conditions to crystallize and grow graphene.

Although the synthesis of graphene faces huge challenges, considerable progress has been made in this field of research in the past ten years or so [106]. Many scholars have devoted themself to the preparation of graphene. The preparation methods can be divided into two categories: graphite exfoliation methods (mechanical exfoliation methods [107], liquid phase exfoliation methods [108], redox methods [109], quenching methods [110], electrostatic deposition methods, etc.) and direct growth methods (vapor deposition methods [111], organic synthesis methods [112], solvothermal methods [113], arc discharge methods [114]) (Figure 6). The interlayer spacing (0.7~1.2 nm) of the prepared graphene is larger than the original graphite interlayer spacing (0.335 nm) [115], which is beneficial to the intercalation of other molecules [116]. Thus, composites of graphene and other materials have been produced and applied in different fields: pollution adsorption, medical antibacterial, fire retardant, thermal conductivity, etc.

### 5.1. Preparation of Mt@GR

#### 5.1.1. Dry-Freezing Method

Dry-freezing is a simple and environmentally friendly method to produce Mt@GR [118]. Its basic principle is heat and mass transfer mechanism at low temperature and low pressure and it almost does not change the volume of the material, maintaining the original structure and avoiding condensation phenomenon. Besides, it can also protect some easily oxidized substances.

For example, Tao et al. [119] added a GO dispersion solution into a Mt suspension and stirred them for 2 h. At the same time, sodium alginate (SA) solution was slowly added in the mixture. After that, the homogeneous solution was added dropwise into CaCl_2_ solution, and then the GO-Mt/SA were repeatedly washed five times, and finally subjected to vacuum freezing for 14 h. Adsorption experiments indicated that the GO-Mt/SA showed a better adsorption capacity towards MB (150.66 mg/g).

#### 5.1.2. Vacuum Impregnation Method

Impregnation methods use a carrier to contain a specific solution impregnation, relying on capillary pressure to add the components into the carrier, while adsorbing on its surface. The principle and operation of vacuum impregnation are basically the same, but the porous material needs to be evacuated first, and then the impregnation liquid was added. Compared with the traditional wet impregnation method, the vacuum impregnation method produces a more uniform and efficient material.

Peng et al. [120] produced stearic acid/reduced graphene oxide modified montmorillonite composites (SA/rGO-Mt) through the vacuum impregnation method. The results showed that energy storage and release rates of SA/rGO-Mt were significantly improved because of the enhanced interfacial thermal transfer by graphene.

### 5.2. Applications of Mt@GR

#### 5.2.1. Pollutant Adsorption

With the continuous development of industrial economy, more and more wastewater containing heavy metal or organic pollutants is being discharged into natural waterbodies. Due to their high toxicity, the increasing discharge volume and the non-biodegradability, heavy metal pollution and organic pollutants in dye wastewater have become one of the main problems endangering human life [121]. Therefore, the development of treatment technology for heavy metal ions in industrial wastewater and organic matter in dye wastewater has received extensive attention from researchers [122].

Compared with traditional chemical methods, physicochemical methods and biological methods, the adsorption method has attracted much attention because of its environmental protection, high efficiency, low cost, and high removal efficiency [21]. Recently, people have tried to develop composite adsorption materials to enhance adsorption performance and solve the problems of poor regeneration and high cost of traditional single adsorption materials.

Due to its unique physical and chemical properties, graphene has received widespread attention as a new type of adsorbent for various heavy metal and organic pollutants, and has become a highly potential adsorbent [123]. However, the layer-by-layer stacking phenomenon of graphene greatly affects its adsorption function. In order to enhance the performance of graphene, it is necessary to improve the dispersion of graphene in the polymer matrix and the interaction between graphene and polymer. Clay minerals represented by Mt are an important part of the soil and are a major adsorbent for heavy metal and organic pollutants [124]. Due to the unique physical and chemical structure of Mt as a natural compounding agent, the composite of Mt and graphene can reduce the layer-by-layer stacking of graphene, and better play the role of adsorbing pollutants.

Adsorption materials based on Mt-graphene composites have been extensively studied by scholars due to their high adsorption performance, low price, and simple preparation process. The composite material, which was made by freeze-drying, hydrazine hydrate reduction, and wet impregnation [125], had good adsorption capacity for rhodamine B (RhB), methylene blue (MB) and the heavy metal ion Ni^2+^ in wastewater.

**Figure 7:**
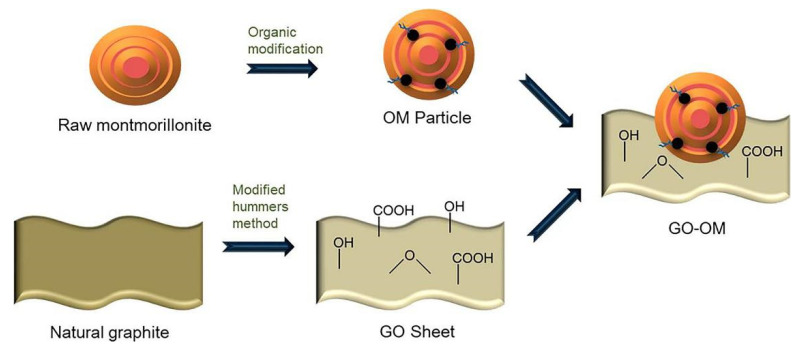
Schematic diagram of GO-OM preparation process [126].

Some scholars have modified natural Mt through organic and inorganic methods to improve the adsorption capacity of composite materials. Wei et al. [126] used the ultrasound-assisted method for the first time to prepare a graphene oxide-supported organic Mt composite (GO-OM) (Figure 7). It showed good synergistic adsorption properties for mixed pollutants in aqueous solution. GO-OM had a very high adsorption capacity for metal cations and oxygen anion groups and showed good potential in removing a variety of pollutants. In addition, this study also revealed for the first time the mechanism of synergistic adsorption, indicating that oxygen and cationic surfactants could promote the synergistic adsorption. Studies have shown that GO-OM has a good treatment effect on a variety of pollutants and is a promising sewage treatment material.

Zhang et al. [127] used metal manganese and graphene oxide as raw materials, and successfully prepared a Mt-graphene oxide composite (MGC) adsorbent through STAC cross-linking. The simultaneous adsorption capacities of MGC for Pb^2+^ and PNP were 19.79 mg/g and 15.54 mg/g, respectively. Compared with Mt and GO, the total adsorption capacity of MG for the two pollutants was significantly improved. However, while preparing materials using metal-containing catalysts, a large amount of metal waste will inevitably occur. Therefore, it is necessary to find a sustainable method to achieve environmental friendliness by improving the recyclability of adsorbent materials. Xiao et al. [128] first prepared spherical Mt (SMt) with a larger specific surface area by spray drying, and then wrapped it with graphene oxide sheets. Gold nanoparticles (Au NPs) were prepared in situ, and SMt@GO@Au NPs microspheres with good adsorption capacity were synthesized by a dopamine chemical method. The prepared micron-sized microspheres could be easily recovered from the catalytic system without any equipment due to its own precipitation effect. After 10 cycles, there was almost no change in catalytic activity and morphological structure, which reflected that Mt@GO@Au NPs microspheres had good recyclability, and the reuse of gold nanocatalysts could be used for large-scale applications of gold and silver. Platinum, copper, and other high-performance metal catalysts provide a way to develop environmentally friendly adsorption materials.

In terms of the adsorption of the greenhouse gas carbon dioxide, Stanly et al. [129] first synthesized a new type of polyphosphoric acid-modified Mt through a cation exchange reaction, and then reduced graphene oxide through in-situ hydrothermal condition. They were hybridized to obtain a PMt/rGO composite material with a surface area of 50.77 m^2^/g and an adsorption capacity of 0.49 mmol/g for CO_2_, which was 42% higher than that of other materials. This showed that clay-based materials represented by Mt were high-efficiency CO_2_ adsorbents. Table 2 shows the adsorption of Mt@GR composite materials on the main heavy metal ions and organic dye pollution in wastewater.

Overall, the data illustrates that Mt, as a typical natural organic compounding agent, can be compounded with graphene to reduce the stacking of graphene layers and increase the actual adsorption capacity of the adsorbent, obtaining better adsorption performance at a lower cost.

#### 5.2.2. Antibacterial Properties

According to reports, the current infections caused by bacteria are still one of the biggest health problems in the world, afflicting millions of people every year [132]. However, the abuse of traditional antibiotics has led to the problem of antibiotic resistance, which makes it extremely difficult to treat infections. People must turn their attention to non-traditional antibacterial materials. In recent years, graphene has been considered as a new type of high-efficiency antibacterial material, which has strong cytotoxic effects on bacteria [131] and fungi [133], and will not lead to the problem of antibiotic resistance. However, its poor solubility and processability severely limit the antibacterial applications of graphene, as it tends to aggregate due to strong inter-plane interactions. Fortunately, this shortcoming can be overcome by adding graphene to the polymer matrix [132].

Yan et al. [134] mixed graphene oxide prepared by the Hummers method [135] with Mt in deionized water at a mass ratio of 1:10 and ultrasonicated them for 8 h at pH = 6~7 to obtain a Mt@GO suspension. Then, they reacted the suspension with copper nitrate, hydrazine hydrate, and finally obtained a uniform Mt-rGO-CuNPs complex. The results showed that the Mt-rGO-CuNPs complex had good antibacterial properties, and the bactericidal effect on *Staphylococcus aureus* (99.43%) was significantly stronger than that on *Escherichia coli* (84.30%).

In order to further improve the antibacterial properties, Wu et al. [136] have successfully prepared cetylpyridinium bromide/Mt-graphene oxide (GM-CPB) composite materials. After 24 h of contact with *Escherichia coli* and *Staphylococcus aureus*, the antibacterial rates of 10 mg/L GM-CPB were 92.3% and 99.9%, respectively. This indicated a great improvement of the antibacterial activity of the composite. In terms of the durability of antibacterial time, many scholars have also explored and tried to improve it. Yang et al. [137] used ascorbic acid as a reducing agent, under the action of polyethylene glycol (PEG) and sodium hydroxide, at room temperature to reduce graphene oxide-supported copper sulfate, and prepared a stable reduction oxidation with long-term antibacterial activity Cuprous (rGO-Cu_2_O) nanocomposite material.

Through the above exploration, it is not difficult to find that, compared with traditional antibiotics, Mt@GR composite has great advantages and potential in the field of antimicrobial medicine.

#### 5.2.3. Flame Retardants

According to worldwide trends, all walks of life are pursuing the use of lighter, thinner, more reliable, and more durable materials, stimulating people’s strong interest in nanotechnology. However, harmful residual products may be produced during combustion, which are not easy to decompose and can cause toxic biological accumulation [138]. Therefore, the research of a non-polluting and efficient flame-retardant material has gradually attracted the attention of scholars.

Inspired by the hierarchical structure of nacre, Ming et al. [139] found that Mt@GO nanosheets can be self-assembled through vacuum-assisted filtration to combine graphene oxide and Mt with PVA and obtain a composite material. These nanocomposites exhibit good flame-retardant properties. In addition, Chen et al. [140] also used a simple green method, similar to papermaking, to prepare a graphene oxide-enhanced wood self-hydrolysate/Mt nanocomposite film, which had high strength, excellent flame retardancy and hydrophobicity. The synthesis method could make the wood self-hydrolyzed liquid be used in the preparation of fireproof film, paint, packaging materials, etc.

Mithilesh and Sharif [141] prepared a new Mt/GO/CS (chitosan) composite film by a simple solution mixing evaporation method, which led to hydrogen bond interactions between chitosan and filler enhanced by Mt and GO. The results proved that the composite film had better thermal stability than chitosan, which further improved the performance of the film. Asgari et al. [142] focused their attention on silane-modified sodium-based Mt, and their research revealed a new result of sodium-based Mt (Na-Mt) silane functionalization: silane-modified sodium-based Mt. Desoil has high thermal stability and is a promising polymer nanocomposite nanomaterial.

It can be seen that the flame retardancy of the abovementioned Mt-GO composite material has been greatly improved, laying the foundation for it to become a new generation of combustion-supporting substances, and also provides a new idea for the research of developing new flame retardant materials.

#### 5.2.4. Thermal Conductivity

Plastic has become one of the most widely used materials in the world, but its inherent structural characteristics determine its low thermal conductivity. Therefore, some studies had been done to enhance the thermal conductivity of plastics. Zhu et al. [143] used Mt/GO as a binary filler, and they then adopted a one-step solution blending method, followed by hydrazine hydrate reduction to prepare Mt/reduced graphene/polyvinyl alcohol (Mt/rGO/PVA) composite film. When the mass ratio of Mt to Mt/rGO_2_/PVA in the composite film was 2:1 and the mass fraction of binary filler was 12%, the thermal conductivity of the composite film reached 66.4 W/(m·K), which was at least 132 times higher than that of pure PVA. The improvement of the structure and composition of the composite film through Mt and rGO could significantly improve the thermal conductivity of the polymer, and it also provided an idea for the preparation of other high thermal conductivity polymer composite films. Liu et al. [144] have solved the problem of low thermal conductivity of the existing water-based impregnating insulating varnishes. They prepared a motor stator through hydroxyl-containing organic amine modified Mt, hydroxylated graphene, and hydroxyl-containing water-based resin. Using Mt@GR hybrid water-based impregnating insulating varnish, the composite material had high thermal conductivity, good permeability, and good water resistance, which overcame the deficiencies in the prior art.

#### 5.2.5. Other Fields

Mt@GR can also be further compounded with other materials for anti-friction additives, lightweight fire-resistant conductors [145], or catalytic organic synthesis [146]. Hao et al. [147] first modified GO with a silane coupling agent to obtain functionalized GO; then, they mixed functionalized GO with natural Mt nanopowder, and thermally reacted with water (or solvent) to obtain a functionalized graphene-loaded natural Mt lubricating oil additive with excellent anti-wear, anti-friction and intelligent repairing effects. And it can reduce the friction coefficient of lubricating oil by up to 32%. Peng et al. [148] synthesized molybdenum disulfide nanosheets on Mt modified with rGO by hydrothermal method, and successfully prepared ternary two-dimensional nanocomposites of Mt, graphene, and molybdenum disulfide. Combining the high conductivity of graphene with the excellent hydrophilicity of Mt can simultaneously improve the electron transfer efficiency and interface reaction efficiency of MoS_2_ nanocomposites, which provides the possibility to construct a new two-dimensional nanoelectrochemical hydrogen evolution composite material performance.

## 6. Preparation and Applications of Mt@Other Two-Dimensional Materials

In addition to LDH and GR, Mt can also be composited with two-dimensional materials, such as MoS_2_, MoSe_2_, BiOCl, MnO_2_ nanosheets [149], etc. As an important application of Mt@other two-dimensional materials, catalysis and pollution adsorption will be discussed.

### 6.1. Preparation Methods of Mt@other Two-Dimensional Materials

#### 6.1.1. Hydrothermal Synthesis Method

Hydrothermal synthesis is a simple synthesis method. Its advantages are high concentration, good dispersibility and easy control of particle size. In the process of hydrothermal synthesis, the samples in aqueous solution are mixed equally at a high reaction rate. In addition, it can form crystalline powder with high purity, so it has been widely used.

Yang et al. [150] synthesized Mt@MoS_2_ by hydrothermal synthesis. Firstly, Mt nanosheets were prepared by ultrasonic exfoliation as the exfoliation of GO. Then Mt was added into deionized water, with stirring and centrifuging to remove coarse particles. Collecting supernatant, further ultrasonic stripping, and centrifuging to obtain supernatant. (NH_4_)_6_Mo_7_O_24_·4H_2_O and CH_4_N_2_S were dissolved in water and added to Mt nanosheet suspension. After halving, the suspension was added into a Teflon-lined autoclave for heat treatment. Finally, Mt@MoS_2_ was obtained via freeze-drying.

The research of Li and Peng [151] also used an in-situ hydrothermal method. In this process, Na_2_SO_4_·2H_2_O, Se, NaBH_4_ were added to deionized water after ultrasonic treatment to produce a uniform solution. Then Mt was added to the above solution and stirred at room temperature. Then, the suspension was transferred to a stainless steel autoclave lined with Teflon-lined stainless-steel autoclave and treated at 200 °C for 24 h. After natural cooling, it was filtered, washed and dried to obtain Mt@MoSe_2_. Rhodamine B was selected for decolorization to evaluate the adsorption performance and photocatalytic ability of Mt@MoSe_2_. It is found that the total decolorization rate can reach 98.2% after 45 min of visible light irradiation.

#### 6.1.2. Ultrasound-Assisted Chemical Precipitation Method

Ultrasound-assisted chemical precipitation methods mainly use the propagation characteristics of ultrasonic waves, increasing the reaction rate. Ultrasonic waves can cause drastic changes in sound pressure, which leads to strong cavitation and emulsifying phenomena in liquids. In a very short time, a large number of tiny air bubbles are generated, and violent blasting occurs continuously. The impact force produced by blasting makes the reaction particles in full contact, achieving the purpose of accelerating the reaction rate.

Mao et al. [152] studied and prepared Mt@BiOCl. Firstly, Bi(NO_3_)_3_·5H_2_O and KCl were dissolved in ethylene glycol, and magnetic stirring was carried out at normal temperature until the solution was clear. Under ultrasonic irradiation conditions, a certain amount of Mt was added to the clear solution to form a suspension. After ultrasonic treatment, distilled water was poured into the suspension system and reacted under magnetic stirring to form a milky white precipitate. The precipitate was filtered by suction, washed with ethanol and distilled water, and dried at 50 °C to obtain Mt@BiOCl. After illumination for 120 min, the degradation rates of catechol and p-aminobenzoic acid were 98% and 92.5%, respectively, indicating that Mt@BiOCl had good adsorption and photocatalysis properties.

### 6.2. Applications of Mt@other Two-Dimensional Materials

#### Photocatalysis and Wastewater Treatment

With the expansion of industrialization, a large amount of organic dye wastewater is produced and discharged every year, which seriously threatens the ecological environment and human health. Photocatalysis with safety and environmental friendliness have been considered as a popular methods to treat organic dye wastewaters. By compounding Mt and other two-dimensional materials with photocatalytic properties, the active sites can be well increased and then the photocatalytic efficiency can be improved greatly.

**Figure 8:**
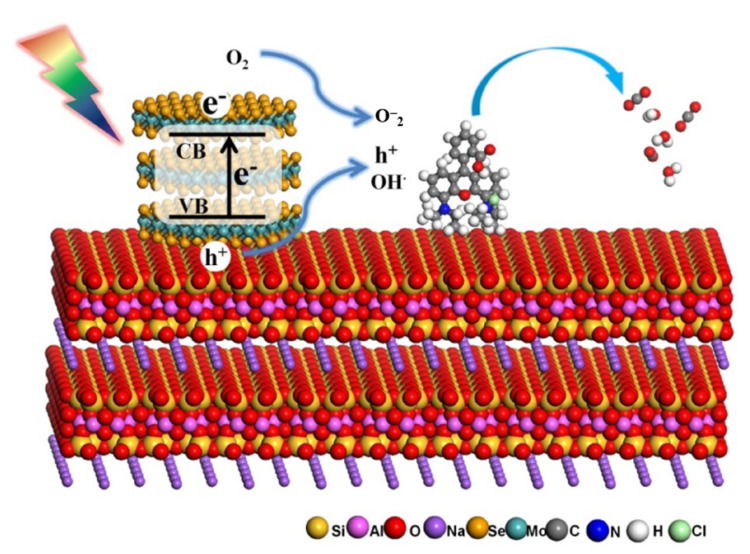
A schematic illustration for the photocatalytic reaction mechanism of Mt@MoSe_2_ [151].

MoSe_2_ is an effective photocatalyst for degrading organic dyes, but its poor dispersion in water and easy aggregation of nanosheets limit its further applications. When MoSe_2_ is compounded with Mt, the aggregation of molybdenum disulfide can be inhibited, improving its dispersibility. Finally, the photocatalyst Mt@MoSe_2_, with high efficiency and low cost, can be obtained [151]. The schematic diagram of Mt@MoSe_2_ photocatalytic reaction mechanism is shown in Figure 8. Another compound with photocatalytic properties is Mt@BiOCl. The Mt@BiOCl photocatalyst can effectively degrade 8-hydroxyquinoline, catechol and *p*-aminobenzoic acid. This reaction follows quasi-first-order reaction kinetics [152]. Besides, the composite catalyst also showed good chemical stability, which could contribute to its practical applications.

Based on the excellent photocatalytic performance of the composite, it is necessary and meaningful to protect the environment using pollution-free and sufficient light energy for sewage treatment. Hence, the combination of Mt and other two-dimensional materials with photocatalytic properties has great potential in catalysis and wastewater treatment.

## 7. Conclusions and Outlook

In this review, we have summarized the preparation and applications of Mt and other two-dimensional materials, and focused on Mt@LDH and Mt@GR. Mt shows strong water absorption, adsorption and good cation exchange performance, but its hydrophobicity and poor compatibility with polymers limit its wider application. At the same time, two-dimensional materials, especially their own active sites, are under-exposed, which makes their functions not well reflected. If the two materials can be compounded, the deficiencies of the two single materials can be overcome. In the preparation of Mt@LDH, there are three general kinds of methods: intercalation methods, in-situ growth methods and tape casting methods. Herein, several methods of delamination are summarized according to the strippers used. Among them, polar solutions of formamide are considered to be the simplest and most effective way. However, because formamide has certain environmental issues, PLA and other environment-friendly stripping agents have been considered to be selected by some scholars. On the other hand, dry-freezing and impregnation methods are also used to prepare Mt@GR. The former is a simple and environmentally friendly method. In the latter method, a carrier containing a specific solution is impregnated, and components enter the carrier by capillary pressure and are adsorbed on its surface. The principle and operation of vacuum impregnation are basically the same, but porous materials need to be evacuated first, and then the impregnation liquid is added. Compared with the traditional wet impregnation method, the materials produced by vacuum aging methods are more uniform and efficient. The preparation of MoS_2_, BiOCl and other two-dimensional materials is also introduced later

## Figures and Tables

**Table 1:** Summary of slice layer stripping method.

Authors	Materials Used	Stripper	Key Features	Strengths	Limitation	Ref.
Zhou et al.	NiAl-LDH	Formamide	Ultrasound conditions	Most effective; dissolves LDHs sheets	Dissolves LDHs sheets	[49]
Hibino et al.	MgAl-Gly LDHs	Formamide	Stir for a few minutes at room temperature	Fast gradual change without heating and reflux conditions	-	[53]
Gong et al.	Nitrate-type magnesium aluminum hydrotalcite modified by sodium dodecyl sulfate intercalation modification	Chloroform	Ultrasound conditions	-	Chloroform is sensitive to light and forms toxic gas when exposed to oxygen	[54]
Dai et al.	CTA-Mt (Organic montmorillonite)	Chloroform	Ultrasound conditions	Chloroform is sensitive to light and forms toxic gas when exposed to oxygen	-	[55]
Liang et al.	LDH intercalated with acetate ion	Water	Ultrasound conditions	Environmental protection, non-toxic, low price	-	[56]
Ma et al.	Synthesis of amino acid intercalated aluminum-magnesium hydrotalcite in a system with pH greater than amino acid isoelectric point	Water	pH is lower than the isoelectric point of amino acids, ultrasound, heating	Environmental protection, non-toxic, low price	-	[57]
Song et al.	Mt	HTPB (hydroxyl terminated polybutadiene)	353 °C	Efficient and can be completely stripped	-	[58]
Hu et al.	Layered dihydroxy compound, Mt	PLA (polylactic acid)	185 °C hydrothermal	Stripping agent is easy to degrade	-	[50]

**Table 2:** Adsorption of pollutants in water by graphene-Mt materials.

Adsorbent	Pollutants	Surface Area(m^2^/g)	Qm (mg/g)	pH	Ref.
Mt-rGO	Ni^2+^	171	625	7	[125]
Mt-rGO	RhB	171	179	7	[125]
GO-OM	Pb^2+^	78	281	7	[126]
GO-OM	Cd^2+^	78	144	7	[126]
GO-OM	As^4+^	78	66	7	[126]
MGC	Pb^2+^	-	20	6	[127]
MGC	PNP	-	16	6	[127]
Mt-GO	MB	75	641	-	[125]
Mt-GO	MB	973	345	9	[130]
Mt-GO	MO	973	132	7	[130]
Mt-GO	Pb^2+^	973	262	-	[130]
GMN	Pb^2+^	-	27	6	[131]

## Data Availability

The data presented in this study are available on request from corresponding author.
